# Conversational repair in individuals with Alzheimer disease

**DOI:** 10.1590/2317-1782/20212021133

**Published:** 2022-04-20

**Authors:** Carolina Martínez Sotelo, Ana Paula Machado Goyano Mac-Kay

**Affiliations:** 1 Escuela de Fonoaudiología, Facultad de Ciencias de la Salud, Universidad de Playa Ancha – UPLA, Valparaíso, Chile.; 2 Escuela de Fonoaudiología, Facultad de Salud, Universidad Santo Tomás – UST, Viña del Mar, Chile.

**Keywords:** Discourse, Speech, Language and Hearing Sciences, Alzheimer Disease, Evaluation, Speech

## Abstract

**Purpose:**

many of the difficulties that usually arise in colloquial conversation may be repaired as the interaction develops. Conversational repair is a linguistic strategy that indicates evidence of the partners‘ abilities to promote necessary mutual understanding for effective communication. This study aims to analyze the oral text repairs of 10 older adults with Alzheimer's dementia (AD) and 10 older adults without dementia, as well as to verify whether they are a useful element for language disorders identification in AD.

**Methods:**

autobiographical interview and application of items of the MetAphAs protocol were proposed. Data were analyzed based on the methodology of conversational analysis.

**Results:**

the results indicated that self-initiated repairs are frequent in both groups, although subjects with AD made more use of inadequate repairs, which did not favor mutual understanding. Hetero-repairs occurred most frequently in conversations with individuals with AD, suggesting the need for interlocutor’s intervention to adjust a specific utterance.

**Conclusion:**

this research has shown that using repair strategies is sensitive to individuals with AD cognitive performance.

## INTRODUCTION

Conversation is a communicative exchange activity that produces a text. The production of the text, within the framework of colloquial orality, supposes the activation of cognitive and linguistic mechanisms that ensure the appropriate logical, semantic, and pragmatic gear. The text is subjected to a constant process of co-construction between the interlocutors who adapt it according to the discursive context^(1)^. Thereby, the speaker must deploy various resources of coherence and cohesion that allow to generate understanding, the expected responses, and a performance adjusted to the purposes determined in the interaction with the interlocutor^(2)^. Context is a mental model built by the speaker and used to establish relationships between general and particular, between personal and social, that is, the speaker links linguistic structures to social situations through the context^(3)^.

Textual formulation is the result of the involved interlocutors’ contributions and constitutes a complex process of negotiation of meanings, where collaborative work is established to achieve mutual understanding^(4)^. Since the exchange is spontaneous, poorly planned and emergent, repetitions and paraphrases abound^(5)^, the appearance of errors, speech or listening failures, interruptions, silences, overlaps etc^(4)^. Related events are also observed by controlling the topic and establishing the reference. Thereby, clarifications, self-corrections and corrections are usually necessary to solve possible difficulties arising, becoming frequent and natural conversational phenomena^(6)^.

In the formulation processes, a set of resources is identified to solve problems of mutual understanding, designated as repair procedures, whose function is to reorganize the discourse under construction in the conversation and try to adapt the intentions, contexts, and proposed meanings for the developing discourse^(7)^. Repair is a frequent linguistic strategy in the interaction and can be carried out by the speaker or by the listener^(7,8)^. It occurs when the thematic development is discontinued in the course of a conversational exchange and is accompanied by other activities, such as gestures of support. It is constituted of a sequence of statements that emerge to repair something said by one or the other interlocutor^(9)^. It is called self-repair when it is speakers themselves perform and repair made by another when it is other initiated. Furthermore, depending on which of the participants has initiated it, one can speak of a “self-initiated repair” and an “other-initiated repair”^(7)^. Self-initiated repair usually appears in the same turn or a later turn, the former being the most common. The other-initiated repair always occurs in the next turn, after the appearance of the problematic element^(7,10)^.

There are four interrelated patterns of repair^(7)^. Self-initiated self-repair: the speaker is responsible for the reparable utterance, and it is he/she who initiates and ends the repair. Self-repair initiated by another: the speaker (is responsible for the reparable statement, and it is the intervention of another participant that causes the speaker to repair. Other-initiated repair derives from the self-initiation: the speaker responsible for the repairable element initiates the self-repair, but it is the other participant who ends it. Other-initiated repair: the speaker is responsible for a repairable element, and it is the other participant in the next turn who initiates and ends the process. Of these four patterns, the most frequent are the first two, observing a greater preference for self-initiated self-repair.

From a point of view of correction opportunities and speaking turns, repairs can occur in the same turn, through a self-initiated self-repair, in the end of the turn, in the transition space (where potentially the first turn ends to give space to the second turn) and in the second turn to carry out a repair initiated by the interlocutor^(10,11)^.

Then, in the course of the conversation, the development of a statement is constantly interrupted in order to make the sequential organization of the discourse coherent, and what is said is correct and accepted by both interlocutors. Understanding that the interruption is the phenomenon that marks the repair, the speaker will have the following alternatives when facing it^(11-13)^: the speaker can return to the interrupted statement by repeating the unit or units that immediately precede the rupture; after an interruption, the speaker takes up the statement with another modifying element. After interrupting the utterance, the speaker makes a subsection introducing another element and then resumes the first with some modifications and the speaker leaves the utterance unfinished.

Authors^(12)^ verified conversational repair in patients with Alzheimer's Dementia (AD) and the results showed that the percentage of significantly higher repair, related to understanding and most of the time successful, was observed in the moderate AD group. The group with mild AD produced more requests for repair than their interlocutors and, in turn, their interlocutors made more repairs in the elaboration of the discourse. Patients with AD use different types of repairs to maintain discursive cohesion and hetero-repairs (made by the patient's interlocutor) fulfill the function of facilitating the overall coherence of the discourse^(2)^. A study^(13)^ investigating the frequency and nature of problems and repairs in conversations between individuals with AD and their interlocutors found that normal interlocutors reported, in a high proportion, moments of collapse in the conversation, assuming a higher burden role as a negotiator of the repair sequence. The subjects with AD, on the other hand, presented more problems related to maintaining the main theme, difficulty of elaboration resulting from the lack of fluency, discontinuity in the conversation, and a greater degree of inadequate repairs. Valles^(14)^ compared this strategy between Broca's aphasia and AD patients and observed that the repair activities differ in conversations between individuals with aphasia and dementia. With the former, a large number of moments of repair are evident, while with the latter, conversational activities are reduced as there is greater cognitive deterioration that contributes to their decrease. The role of the interlocutor also differs, as follows: aphasic individuals seek to repair more the form to achieve adequate cohesion, while patients with dementia use indirect repairs seeking to repair global coherence. An analysis of repair activities in Brazilian aphasic patients concluded that these discursive movements constitute a process of reconstructing meaning in the presence of linguistic deficits (such as paraphasia, difficulties in accessing the lexicon etc.)^(8)^.

Martínez and Noemi^(15)^ analyzed the conversational repair activities in three groups of participants identified as controls, with mild cognitive impairment (MCI) and Alzheimer's dementia (AD), and in a conversational elicitation activity based on a cartoon in pictures. It was observed that the control subjects carried out conversational repair activities, appropriate to the course of the conversation through syntactic or lexical corrections. In addition, difficulties in accessing the lexicon were among the causes of the need to repair. On the other hand, the speakers with MCI presented errors in character recognition, actions or motivations, inattentions, incoherencies, and difficulty in staying on the topic. What stood out in this group is that not all repair activities fulfilled the objective of repairing what was previously said to adapt the speech or favor mutual understanding, essential aspects in developing a conversation. Thereby, it can be evidenced the effect of underlying pathology on communication. In the group with AD, the repairs were less frequent and none of the repair sentences completed the objective of adapting the speech for mutual understanding. The authors suggest that the lack of repair activities may have resulted from to the decline or absence of meta discursive assessment and self-monitoring procedures, as well as that the ability to perform self-repairs decreases as cognitive performance declines. Frequent activity in the interlocution with speakers with MCI and AD was of hetero-repair nature. This data evidence the fact that adjusting the conversational discourse, in these cases, is the interlocutor’s responsibility, who needs to be trained for such.

The repairs carried out by subjects with aphasia and dementia in conversations as a couple were compared with a focus on the interactive and communicative aspects^(16)^. The results indicated that the repair sequences were more frequent in those with dementia, in addition to showing the active role of healthy interlocutors in problem-solving. An investigation^(17)^ analyzed the sequential patterns of behavior related to the manifestation of deficiencies in the management of issues and facilitation behaviors in everyday interactions between individuals with dementia and their family members. It was observed that difficulties in contributing to the issue of conversations were dominant in individuals with AD; in addition, the family members applied two types of repair strategies: the former offering an explicit start of a repair, and the latter making topic changes that function as a bridge towards the return to the topic of the previous turn.

Experiences with older individuals with different cognitive conditions allow exemplifying the usefulness of assessing targeting the conversation, as well as examining the relationship among diagnosis, cognitive performance, and linguistic and conversational abilities^(18)^. The Conversational Analysis (CA) revealed a multiplicity of elements such as the functioning of communication in a real context, in a non-idealized way but based on data, analysis of the conversational discourse for its content and not exclusively for what it does not contain^(6)^. The goal of this research was to analyze repair activities in older individuals with and without AD through CA, conversational aiming to learn whether identifying this phenomenon when assessing an individual with AD is useful for diagnosis and/or intervention.

## METHODS

This study followed the approach of descriptive anti-qualitative methodology. The qualitative description orientation focuses on the process rather than on obtaining results, therefore, the conclusions are not generalizable, but rather oriented to the particular phenomenon. The research was approved by the Centro Norte-UST scientific ethics committee nº142 / 2017, on February 28, 2018, assigning the code 153.17, and all participants or their legal representatives signed an informed consent form.

### Sample

of the study involves a convenience sample of older adults or residents in a long-stay private establishment for elderly individuals (LSEOA) of the V Region of Chile, voluntarily available and accessible upon meeting the established inclusion and exclusion criteria. 20 subjects were selected and divided into two groups: 10 in the control group (CG) and 10 in the group of users with Alzheimer dementia (ADG). The following inclusion criteria were applied to the CG: individuals older than 65 years, both genders, presenting a level of education of more than 4 years, use of a hearing aid in case of mild to moderate presbycusis, result in the MEEM and Pfeffer within the cut score described in the test norms, and no history of neuropsychiatric diseases. The following exclusion criteria were applied: presenting neurological or psychiatric diseases at different severity degrees, presenting severe hearing loss and consuming drugs (psychotropic) that influenced the performance of the cognitive assessment. The following inclusion criteria were applied to the ADG subjects: individuals over 65 years of age, both genders, presenting a level of education of more than 4 years, use of a hearing aid in case of mild to moderate presbycusis, current neurological or geriatric medical diagnosis of Alzheimer's dementia (AD) at grade 5 according to the Global Deterioration Scale (GDS) (moderate dementia), meet the DSM-V criteria for major neurocognitive disorder and the clinical criteria of the National Institute of Aging (NIA), and/or the central clinical criteria for probable AD in sections A and C established by the Alzheimer's Association (AA). The following exclusion criteria were adopted: presenting neurological diseases such as stroke or head trauma, psychiatric condition, evidence of comorbidity of another neurological or non-neurological disease affecting cognition, consuming drugs (psychotropic) that could influence the cognitive assessment performance, and a diagnosis of severe hearing loss.

It is worth highlighting that in the analysis of daily conversation, it is often enough to use a sample with a few texts – no more than ten interviews – in order not to generate the phenomenon called 'theoretical saturation' that accounts for the information redundancy in the data, a situation that does not contribute significantly to theoretical reflection^(19)^.

### Procedures

The following neuropsychological tests were applied to each group to assess their cognitive state: Mini Mental State Chile version (MMSE), Abbreviated Boston Test, Frontal Assessment Battery (FAB), FAS, and semantic verbal fluency test (animals and actions), in addition to Weschler's direct and inverse digit span, King's auditory-verbal memory test, and Pfeffer Questionnaire. Clinical elicitation was the technique chosen for data collection^(20)^ which implies that the conversation is provoked by the researcher and then develops as naturally as possible. Two conversational tasks were used to generate a corpus, which was considered sufficient for each subject, encompassing two tasks: conversational task 1 - semi-structured autobiographical interview, with open questions answered by each participant from their perspective, without informative clues, according to their rhythm and natural adaptations within the discursive context. Conversational task 2 – composed of the following items from section VI of the MetAphAs Protocol^(21)^: item 31, description of an absent object or situation; Item 32, time displacement I-the participant is asked to tell us about what they did on the weekend; Item 33, time displacement II: the participant must tell about their first job, what it consisted of, and their activities; Item 34, temporal displacement III: the participant is asked to tell what they intend to do next weekend or on the next vacation; Item 35, interpret a scene; Item 39, ability to lie: the participant is asked to tell a simple lie, and item 40, ability to be ironic: the participant is asked to be ironic about something based on an example. The MetAphAs Protocol was chosen for being a metalinguistic assessment instrument related to the nature of conversational repair and applied ecologically in nature, favoring the development of a natural conversational context.

CA is used in this study, which is among the main streams of ethnomethodological research^(22,23)^, whose approach is to analyze empirical data from natural texts in order to discover the mechanisms through which the actors give meaning to what happens to them, their expressions, and their own actions. The data were transcribed using the list of conventions adapted by Tusón^(24)^ and the transcripts were transferred to the AtlasTI version 7 software, including the presence or absence of repair activities, types of sources of problems, type of repairs, and results of the conversational repair activity. The quantity, frequency and conversational linguistic context in which these activities appear were also analyzed.

As there is no single way to carry out the analysis procedure, the following aspects were considered as a coding paradigm: (a) open coding: analytical process by means of which the concepts are identified, and their properties are discovered in the data. Dimensions and their coded, that is, the code found is conceptualized (for example, in the text, a question by the participant can be marked as “what?”, which can be coded as “request for clarification”, which is a request for signal clarification of what is being said); (b) axial coding: a process in which the codes found in the previous process are classified, arranging them into categories that favor the analysis of meaning connections of the verbal elements, their consequences, derivations, and interpretations to break down the phenomenon of repair to find characteristics, rules of use, properties etc.; (c) selective coding: the last stage where the theory is integrated and refined, systematizing the data that will account for the knowledge produced from the observation of repair activities, thus answering the research questions^(20)^. The corpus was built from the conversations, on which the coding, the generation of concepts and the development of explanations from the same data were carried out, which were then contrasted with the previous theoretical framework.

## RESULTS

Below, Table 1 presents the characteristics of the CG and ADG .

**Table 1:** Characteristics of CG and ADG according to gender, education, and age

	**Code**	**Controls**		**AD**	
		**Freq.**	**Percent.**	**Freq.**	**Percent.**
**Gender**	Male	3	30	2	20
	Female	7	70	8	80
**Education**	<8	2	20	2	20
	8- 12	7	70	6	60
	>12	1	10	2	10
**Age**	66-69	1	10	1	10
	70-74	5	50	0	50
	75-79	2	20	1	20
	80 and +	2	20	8	80

The data on the individuals of both groups are similar in terms of gender and education. For to age, 80% of ADG are 80 years old or over, which is consistent with the late onset of persisting time of AD dementia^(25)^.

Table 2 shows the results of the neuropsychological assessment performed in the individuals of both groups.

**Table 2:** Results of the neuropsychological tests of the participants in the control and case groups, mean, standard deviation, and Student's t-test

**Test**	**Group**	**N**	**A**	**SD**	**T-Student Test**
**MMSE**	**CONTROL**	10	27.30	2.983	0.000
	**AD**	10	14.90	6.154	
**PFEFFER**	**CONTROL**	10	0	0	0.000
	**AD**	10	22.70	7.072	
**FAB**	**CONTROL**	10	13.60	2.716	0.003
	**AD**	10	8.10	4.149	
**FVSEM Animals**	**CONTROL**	10	11.40	3.658	0.001
	**AD**	10	5.60	2.503	
**FVSEM Actions**	**CONTROL**	10	10.50	5.968	0.032
	**AD**	10	5.40	3.534	
**FVF-F**	**CONTROL**	10	8.90	3.315	0.000
	**AD**	10	2.80	2.486	
**FVF-A**	**CONTROL**	10	8.60	4.351	0.021
	**AD**	10	3.90	3.957	
**FVF-S**	**CONTROL**	10	8.20	2.741	0.013
	**AD**	10	4.50	3.240	
**Ravalt- Trail 1**	**CONTROL**	10	4.20	0.789	0.010
	**AD**	10	1.80	1.619	
**Ravalt- Trail 5**	**CONTROL**	10	7.10	3.178	0.030
	**AD**	10	3.10	1.729	
**Ravalt- Post Intef.**	**CONTROL**	10	5.00	2.261	0.010
	**AD**	10	1.80	1.317	
**Ravalt- Rec Diferido**	**CONTROL**	10	4.60	2.875	0.000
	**AD**	10	0.50	0.972	
**Ravalt-Reconoc.**	**CONTROL**	10	7.80	1.476	0.000
	**AD**	10	2.10	1.197	
**SPAN DD**	**CONTROL**	10	4.40	0.516	0.210
	**AD**	10	3.90	1.101	
**SPAN DI**	**CONTROL**	10	3.30	0.675	0.027
	**AD**	10	2.10	1.370	

Mini mental (MMSE), Pfeffer questionnaire, Frontal assessment battery (FAB), phonemic verbal fluency tests (FAS) and semantics (animals and actions), King's auditory-verbal memory test (RAVLT, test 1, test 5, post interference, delayed recall and recognition, Span Direct Digits (Span DD) and Span Indirect Digits (SPAN ID).

The data show that the CG achieves higher scores and with typical performance in relation to the ADG, with results that classify the individuals as cognitively impaired^(26-28)^. It is worth noting that regarding the levels of functionality from the Pfeffer scale, the scores of four of the resident participants in LSEOAs are lower, probably because as a general rule they cannot comply with daily activities, such as cooking and bathing, since they must always be assisted, regardless of their cognitive or functional level. The t-Student test shows a significant difference in both groups in all tests except in the digit span where the control group achieves a low normal performance.

**Figure 1:**
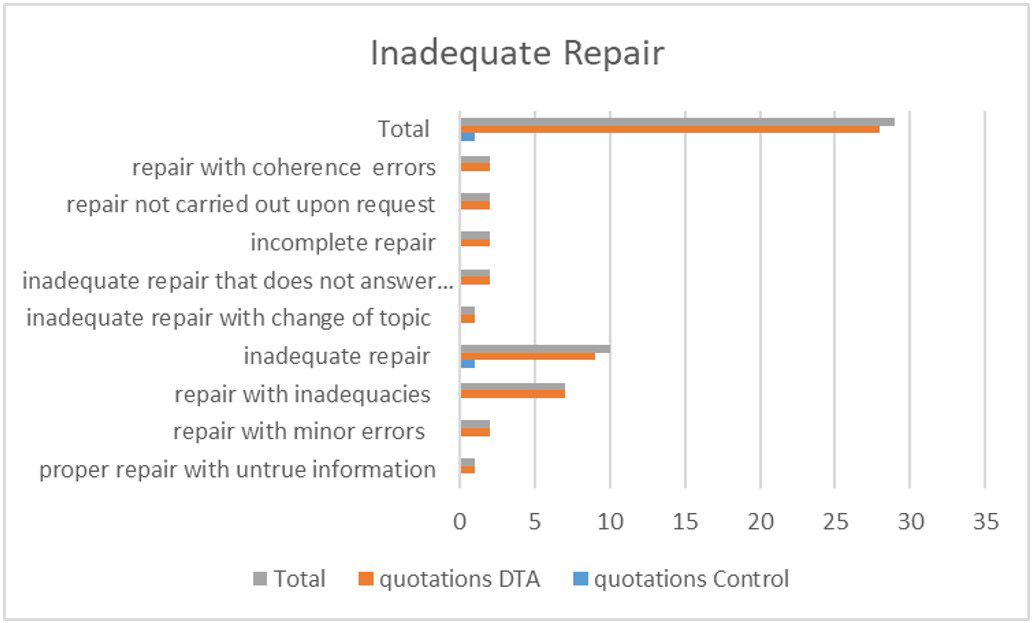
Number of citations associated with inadequate repair in the control and AD groups. Legend: repair with coherence errors; repair not carried out upon request; incomplete repair; inadequate repair that does not answer the question; inadequate repair with change of topic; inadequate repair; repair with inadequacies; repair with minor errors; proper repair with untrue information

Regarding the corpus analysis, we proceeded with the open coding process, and then axial coding where categories and subcategories were related and the codes regarded as the most relevant were selected to form the thematic focuses of analysis. Table 3 refers to the number of citations of repairs and shows that the most uneven data between the groups are related to inappropriate repairs. Figure 1 shows the number of citations of the types of inappropriate repairs.

**Table 3:** Number of citations associated with self-repairs in the control and AD groups

**CODES**	**CONTROL APPOINTMENTS**	**AD APPOINTMENTS**	**TOTAL**
**Self-initiated self-repair**	80	70	150
**Hetero-repair**	10	19	29
**Adequate repair**	94	81	175
**Inadequate repair**	1	28	29

## DISCUSSION

The following is a description of the most frequent codes and a discussion of their importance in the study context. Chart 1 brings together the predominant types of repairs.

**Chart 1:** Types of repair

Self-initiated self-repair
Hetero-repair
Adequate repair
Inadequate repair

In the examples, each of the speakers is identified as 'E' – referring to the interviewer –, 'P' – referring to the participant, and 'A' – referring to the companion. P is associated with a number used to identify the participant and their interview, 'S' is added to indicate if the individual is 'healthy' and 'D' to indicate a participant of the ADG, and a number is added in the end indicating the position of the citation in the text.

### Self-initiated self-repair

The analysis indicated that self-initiated self-repair was the most frequent code in the entire corpus, accumulating a total of 150 citations. Of these, 69 belonged to ADG interviews, while 81 citations belonged to CG interviews. The fact that this type of repair is frequent and dominant throughout the corpus coincides with the literature reports^(7,14,16)^.

Example 1:

P: That whole week I was where my grandson | then || I had to come to, **I had to come back to La Ligua

(PS20: 078)

In this example, the self-initiated self-repair can be clearly seen (the double-asterisk marks the start of the repair that replaces what had been said). In the following quote, the subject clearly self-repairs by changing the expression 'for Christmas' to 'Christmas Eve'

Example 2:

E: they did so? | already and: now who do you live with?

P: not if I live with anyone\

E: here with anyone?

P: sure

E: already:

P: I'm alone here | a nephew is coming now like for christmas\ **Christmas eve | he came to give me the gift\

(PD2:95-100)

### Hetero-repair

Hetero-repair was less frequent than self-repair. The total number of citations was 29, out of which 20 corresponded to the ADG, and 9 to the CG. The difference between both groups could be explained as a phenomenon associated with the presence of linguistic alterations, where the user with AD requires more support from their interlocutor and has more difficulty in performing metalinguistic evaluations; therefore, relying on the interlocutor favors the continuity of conversational interaction^(2,12,13,29)^.

Example 3 demonstrates the user’s difficulties in saying what her job was like as a child, which consisted of delivering lunches to workers. The interlocutor summarizes what has been said to facilitate mutual understanding and the conversation to progress.

Example 3:

P: the workers to go take a bone bakery to a pharmacy that had with: *it was one first on twelve, then one that was another group and so on\until 1:30*

E: **already\ to several groups per turn\ (PD6:027-028)

Another emerging element is marked with the code “reaffirmation of repair”, which is an action that closes the hetero-repair and fulfills a no less relevant function. Although it is not as frequent, its appearance was more or less similar in both groups. As a phenomenon, it can account for the connection between the speaker and what is being said, or at least indicating by their acceptance that they are still present in the communicative interaction^(7,15)^. The code had 11 citations and 5 of them correspond to users with AD.

In example 4, the participant with AD should mention the city where she lived and makes a mistake mentioning one of the main streets. After the interviewer’s hetero-repairs, the participant accepts and validates the correction, and from there she can continue with what she was saying.

Example 4:

*P: in Condell* who was at that time

E: **in Valparaíso

P: If that is | Then we lived there in Valparaíso

(PD4:108-110)

Subsequently, we offer the example of the corpus of a participant with AD with GDS 5, whose speech, often unintelligible, reveals various marks of lack of coherence, failing to present a connection with new topics. She tries to tell us about her first job and when she tries to say that her grandmother prepared lunches, she says, “she arranged” – the interviewer repairs by saying “she prepared them” and she responds by saying “sure.” Her statement does not necessarily indicate that she understood in the context of E's intervention, but rather indicates that she marks her presence in the conversation. It is interesting to note that by responding, she reveals that her ability to interact conversationally is preserved.

Example 5:

E: already: and after the first time I work?

P: I was going to take lunch to school \ | but later I would upload the | the legs that I had to return

E: already: | you gave him food \

P: no: I didn't give it my grandmother gave it everything |

E: already:\

P: | in other words she: | *arranged*

E: **= prepared them =

P: sure

(PD14:043-049)

### Types of repair sentences

A total of 204 citations were marked in the corpus divided into different types of repair statements, which has already been reported in the literature^(14,15,17)^. The three most common types are described below.

- *Repair statement of type syntactic and semantic extension* adds information by a syntactic and semantic extension compared to the source statement. A total of 42 citations are concentrated in this code, 19 corresponding to ADG interviews and 23 to CG.

Example 6:

Q: I was good **when I was in the regiment I was in Punta Arenas

(PD1:025)

In this example, the repair sentence repeats “I was” and adds more information expressed in a sentence with more syntactic and semantic elements, in such a way that it completes information in relation to what the participant wishes to say.

In the next excerpt, the participant adds more information to the repair sentence compared to the source sentence.

Example 7:

Q: (…) | and I'm coming here | **Saturday I would come here and come to my house | (…)

(PS20:052)

The slight difference in the number of citations for this code could suggest that healthy participants are more able to linguistically expand the information than participants with AD, since metalinguistic activity – part of metacognition – regulates the linguistic possibility to make use of subcodes, both oral and written^(17)^.

### *Repair statement of informative correction*. This code includes 51 citations with 36 corresponding to the ADG interviews and 15 to healthy individual interviews.

Example 8:

E: and who do you live with now?

P: | I live alone\

E: here?

P: **I can't live here with all the people

(PD5:105-108)

In this example, the participant with AD is wrong to say who she lives with. Although she corrects it through a repair procedure, the new statement is not as accurate as it does not inform that she lives in an LSEOA. In the following example, we can see how the healthy participant corrects what was said above to make sense of the story she is telling.

Example 9:

P (…) | and currently, not currently, ** like 25 years ago, she had a tremendous business in Viña (...)

(PS23:396)

This code demonstrates a significant difference in the number of citations per group. Participants with AD apparently toned to correct statements more than healthy participants for reasons of cognitive problems (2,14,15,18).

- *Repair statement of informative precision*. This code accumulated 50 citations with 12 citations corresponding to individuals with AD, and 38 to the CG

Example 10:

Q: | I had my house | they lived in the 2 story house\

E: m:

P: In other words**that the bottom part is leased and I lived upstairs (...)

(PD2:024-026)

Example 11:

P: that is pure dangerous circumstances and | and the father was washing the dishes | he is drying a glass | but he has a | full of foam wash | **dishwasher

(PS22:029)

In the case of example 10, the speaker repairs to make the information about where she lived before entering LSEOA more precise. In example 11, the speaker repairs it by making the information more precise in the context of describing a picture.

The notable difference in the number of citations of both groups suggests that for the DG, the repair has the purpose of adding greater precision in what is wished to be said for a better understanding. If we also take the repair code of informative correction as a reference, we can see that the participants with AD are more concerned with correcting than with being more precise. The concern for precision is more rhetorical, indicating that the speaker is constantly monitoring and collaborating on mutual understanding, a fact that is reduced in speakers with AD^(12-15)^.

### Appropriate repair

In the appropriate repair the function of making the statement more understandable and acceptable and facilitating mutual understanding is evident. Its aim is to assess the quality of the repair in terms of whether it repairs or not and favors mutual understanding or not. A total of 175 appointments were marked, with 83 belonging to participants with AD and 92 to control participants. It is worth mentioning that the 92 citations correspond to the total number of repairs observed in healthy subjects, that is, all repairs (self-repair and hetero-repair) performed by healthy individuals were adequate.

Example 12:

Q: but I *knew* the: | the **the place where my father was born Punta Arenas was from Puntarenas

(PD1:025)

Example 13:

P: no, no, what happens is that my lady got sick:: **fell here in the bedroom and broke her hip

(PS23:035)

In the example above, the repair adapts what is said to the interlocutor effectively. The same can be seen in Example 13, taken from an interview with a healthy participant.

The corpus of the sample includes several repair activities, and the main difference between the groups is that the number of repair activities in the ADG is greater; in addition, not all of its repairs are adequate. The repairs marked as adequate were similar to those of the CG, however, the reasons for repair varied since lexicon access difficulties, memory problems and other typical AD alterations that motivate to repair to appear occurred in the conversational interaction . It is also worth considering that 23% of the adequate repairs in the ADG derive from hetero-repairs where the responsibility of adapting what is said relies on the healthy interlocutor.

### Inadequate repair

An inadequate repair is performed by the speakers, but by understanding the impossibility of repairing, clarifying or improving what is being said. The total of citations was 27 and all were verified in the interviews with participants of the ADG.

Example 14:

P: (…) my grandmother had me ready *and I was leaving the beach* (gesture with a mistake head) that is, **the school beach (…)

(PD6:62)

In this example, the participant tries to tell us about the moment when she started her work, 'when she left school', then, a paraphasia 'beach' appears, she manages to realize her mistake, initiates a self-repair, but cannot omit from her statement the word 'beach'.

A repair statement usually appears in the speech marked with a marker or indicator (for example: that is, that is, rather, I say etc.), which allows establishing a semantic relationship with the source statement^(15)^. In this way, repair procedures appear after the speaker has evaluated the previous expression as insufficient, inappropriate, or unsatisfactory. A relevant aspect observed in CG speakers is that the result of the conversational repair activity was identified as adequate, which is the main objective of any type of conversational repair. Such a procedure was less frequent or simply did not occur in the speakers of the ADG group. Although having carried out repair activities with a similarly to the CG subjects, they mostly did so also due to other types of difficulties, including lexicon access, elaboration of statements, incoherent sources, paraphasia events, narrative errors, and marked difficulty in staying on the topic. This performance is in accordance with literature reports on low cognitive performance as a factor of linguistic difficulties (which, in turn, generates conversational repair activities)^(14,15,18,29,30)^.

The presence of inappropriate repair statements or unable to meet the objective of adjusting the discourse or promoting mutual understanding characterizes the degree of adequacy of the ADG. These data confirm the conclusions of authors^(12-16,29)^ who found lower ability to carry out context-appropriate repairs in individuals with mild cognitive disorder and dementia. In the ADG, the adaptation could be achieved mainly through hetero-repairs performed by a healthy interlocutor and allowing to repair the meaning of what is being said. The reduction of self-repair observed in this group may be due to the decrease or absence of meta discursive evaluation procedures^(16,18)^, which is related to the cognitive monitoring of the speakers during the conversation^(18)^. Therefore, by having reduced cognitive performance, the ability to repair is either low or null.

In summary, self-initiated self-repair was observed on a smaller scale and hetero-repair in a higher proportion in individuals with AD comparing with healthy individuals. The repairs analyzed in the ADG showed frequent difficulties in accessing the lexicon, memory, and comprehension. In terms of repair activities, it was consequently possible to observe source statements with errors, either imprecise or incomplete.

70% of the CG are between 70 and 79 years old, showing a significant difference between both groups. Such result reveals that the age difference could have some implications in relation to cognitive and conversational performance, under the premise that aging generates cognitive and communicative changes in older subjects. However, it is important to bear in mind that age does not reflect proportionally the deterioration degree that a person may have, since aging is characterized by its significant inter-subject variability. For this reason, scores obtained in neuropsychological tests allow to interpret the real cognitive state of an elderly person, generating data that allow to compare individuals and groups of individuals. This information would not be interpretable based only on age. Regarding the implications at the conversational level, to date, there is very little evidence on the relationship between age and the ability to repair (Martínez and Noemi, 2016; Mac-Kay, et al, 2017). For this reason, the main findings of this study are especially related to the cognitive level of the participants, considering that age should be further addressed in prospective conversational performance in elderly adults.

## CONCLUSION

This research demonstrates that the capacity of conversational repair is sensitive to the cognitive performance of individuals with AD. The adequacy degree is the main factor of difference between the two groups. The data indicated a decrease in the ability to repair, evidenced in speakers with AD who manifest themselves with repairs that did not meet the objective of correcting or adapting the speech or were inappropriate. Another important result is related to the functionality of the conversational interaction since it was not necessarily sustained in due time in the ADG discourse. It is worth emphasizing that the interlocutor plays an important role in the dyad by trying to compensate for the limitations of the person with dementia.

It is also worth highlighting the importance of conducting further studies on conversational skills and repair activities in the context of healthy elderly individuals, as well as those with pathologies that affect cognitive-communicative functions.
