# Supplementary material for: Conversational repair in individuals with Alzheimer disease
Source: Codas. 2022 Apr 20;34(5):e20210133. doi: 10.1590/2317-1782/20212021133 (PMC9886176; doi:10.1590/2317-1782/20212021133)
Supplement: Translation

# Translation: Reformulación conversacional en personas con la enfermedad de Alzheimer

**Authors:** Carolina Martínez Sotelo, Ana Paula Machado Goyano Mac-Kay

**Affiliations:** 1 Escuela de Fonoaudiología, Facultad de Ciencias de la Salud, Universidad de Playa Ancha – UPLA, Valparaíso, Chile.; 2 Escuela de Fonoaudiología, Facultad de Salud, Universidad Santo Tomás – UST, Viña del Mar, Chile.

**Keywords:** es, Discurso, Fonoaudiología, Demencia de Alzheimer, Evaluación, Habla

## Abstract

**Objetivo:**

en la conversación coloquial surgen diversas dificultades que se van reparando en vivo mientras esta se va desarrollando. La reformulación conversacional es una estrategia lingüística que va dando cuenta de las capacidades de los interlocutores para favorecer la comprensión mutua necesaria para la comunicación efectiva. El presente trabajo tiene como objetivo analizar las reformulaciones de 10 adultos mayores con Demencia tipo Alzheimer (DTA) y 10 adultos mayores sin demencia, y determinar si constituyen un elemento útil para el diagnóstico de la persona con DTA.

**Método:**

se eligió el análisis conversacional como procedimiento metodológico y se propuso una entrevista de tipo conversación de corte autobiográfico y la aplicación de algunos ítems del protocolo MetAphAs.

**Resultados:**

los resultados muestran que la reformulación auto-iniciada es más frecuente que las demás en ambos grupos, aunque las personas con DTA realizan mayor número de reformulaciones inadecuadas que muchas veces no favorecen la comprensión mutua. La hetero-reformulación aparece con mayor frecuencia en las conversaciones en personas con DTA, indicando la necesidad de la intervención del interlocutor para reparar la conversación.

**Conclusión:**

en la presente investigación se ha podido evidenciar que la capacidad de utilizar la estrategia de reformulación es sensible al desempeño cognitivo de personas con DTA.

## INTRODUCCIÓN

La conversación es una actividad de intercambio comunicativo que produce un texto. La producción del texto, en el marco de la oralidad coloquial, supone la activación de mecanismos cognitivos y lingüísticos que velan por el adecuado engranaje lógico, semántico y pragmático. El texto pasa por un constante proceso de co-construcción entre los interlocutores que lo van adaptando según el contexto discursivo^(1)^; así, el hablante debe desplegar diversos recursos de coherencia y cohesión que permitan generar la comprensión, las respuestas esperadas y una actuación ajustada a los fines determinados en la interacción con su interlocutor^(2)^. El contexto es un modelo mental que el hablante ha construido con el que puede establecer relaciones entre lo general y particular, entre lo personal y social, es decir, el hablante a través del contexto enlaza estructuras lingüísticas a situaciones sociales^(3)^.

La formulación textual es el resultado de los aportes de los interlocutores involucrados y constituye un proceso complejo de negociación de significados, en donde se establece un trabajo colaborativo para alcanzar la comprensión mutua^(4)^. Siendo el intercambio de naturaleza espontánea, poco planificada y emergente, abundan las repeticiones y las paráfrasis^(5)^, la aparición de errores, fracasos de habla o de escucha, interrupciones, silencios, solapamientos, etc^(4)^. Se observan también eventos relacionados con el control del tema y establecimiento de la referencia y, por consiguiente, aclaraciones, autocorrecciones y correcciones suelen ser necesarias para resolver posibles dificultades decurrentes, constituyéndose en fenómenos conversacionales frecuentes y naturales^(6)^.

En los procesos de formulación se identifican un conjunto de recursos para resolver problemas de mutua comprensión, designados como procedimientos de reformulación (*repair*), que tienen por función la reorganización del discurso en construcción en la conversación y procura adecuar las intenciones, los contextos y los sentidos propuestos para el discurso que se está haciendo^(7)^. La reformulación. que es una estrategia lingüística frecuente en la interacción y que puede ser realizada por el hablante o por el oyente^(7,8)^, ocurre cuando se discontinúa el desarrollo temático en el curso de un intercambio conversacional, va acompañada de otras actividades, tales como los gestos de apoyo y está conformada por una secuencia de enunciados que pasan a reparar algo dicho por uno u otro interlocutor^(9)^. Se denomina auto reformulación cuando es el hablante mismo que realiza y reformulación hecha por otro cuando es realizada por el otro; además, dependiendo de quién de los participantes la ha iniciado, se puede hablar de una “reformulación auto-iniciada” (*self-initiated repair*) y de una “reformulación iniciada por la otra parte” (*other-initiated repair*)^(7)^. La reformulación auto-iniciada suele aparecer en el mismo turno o en uno posterior, siendo el primero caso el más común. La reformulación iniciada por el otro ocurre siempre en el turno siguiente, después de la aparición de elemento problemático^(7,10)^.

Existen cuatro patrones de reformulación relacionados entre sí^(7)^: auto-reformulación auto-iniciada: el hablante es responsable del enunciado reparable y es él/ella quien inicia y termina la reformulación; auto- reformulación iniciada por otro: el hablante^(^ es responsable del enunciado reparable, y es la intervención de otro participante lo que provoca que el hablante realice la reformulación; reformulación por parte del otro derivada del auto-inicio: el hablante responsable del elemento reparable inicia la auto- reformulación, pero es el otro participante quien la termina; reformulación por parte del otro: el hablante es responsable de un elemento reparable y es el otro participante en el siguiente turno quien inicia y termina el proceso. De estos cuatro patrones, los más frecuentes son los dos primeros, observándose una preferencia mayor por la auto-reparación auto-iniciada.

Desde un punto de vista de oportunidades de corrección y turnos de habla, las reformulaciones pueden darse en el mismo turno, mediante una auto-reparación auto-iniciada, al final del turno, en el espacio de transición (en donde potencialmente termine el primer turno, para dar espacio al segundo turno) y en el segundo turno para dar curso a una reparación iniciada por el interlocutor^(10,11)^.

En el transcurso de la conversación, entonces, el desarrollo de un enunciado se ve constantemente interrumpido con la finalidad de hacer que la organización secuencial del discurso sea coherente, y que lo dicho sea correcto y aceptado por ambos interlocutores. Entendiendo que la interrupción es el fenómeno que marca la reformulación, frente a ella el hablante tendrá las siguientes alternativas^(11-13)^: el hablante puede retomar el enunciado interrumpido repitiendo la unidad o unidades que preceden inmediatamente a la ruptura; el hablante tras una interrupción retoma el enunciado con otro elemento haciendo una modificación; el hablante después de interrumpir el enunciado hace un inciso introduciendo otro elemento para después retomar el primero con algunas modificaciones y el hablante deja el enunciado sin acabar.

Autores^(12)^ verificaron la reformulación conversacional, en pacientes con Demencia Tipo Alzheimer (DTA) y los resultados mostraron que el porcentaje de reparación significativamente mayor, relacionada con la comprensión y la mayoría de las veces exitosa, se observó en el grupo DTA moderado. El grupo con DTA leve produzco más solicitudes de reformulación que sus interlocutores y, a su vez, sus interlocutores realizaron más reformulaciones en la elaboración del discurso. Los pacientes con DTA usan distintos tipos de reformulaciones para mantener la cohesión discursiva y las hetero- reformulaciones (hechas por el interlocutor del paciente) cumplen la función de facilitar la coherencia global del discurso^(2)^. En un estudio^(13)^ que investigó la frecuencia y naturaleza de los problemas y reformulaciones en las conversaciones entre personas con DTA y sus interlocutores y se verificó que los interlocutores normales señalaron, en una alta proporción, los momentos de colapso en la conversación, asumiendo un rol de mayor carga como negociador de la secuencia de reparación. Los sujetos con DTA, en cambio, presentaron más problemas que se relacionaron con el mantenimiento del tema principal, dificultad de elaboración resultante de la falta de fluidez, discontinuidad en la conversación, mayor grado de reparaciones inadecuadas. Valles^(14)^, comparó esta estrategia entre pacientes con afasia de Broca y pacientes con DTA, y se observó que las actividades de reformulación son distintas en conversaciones entre personas con afasia y demencia. Con los primeros se evidencia gran cantidad de momentos de reformulación, mientras que con los segundos las actividades conversacionales se reducen pues hay mayor deterioro cognitivo que concurre para su disminución. El rol del interlocutor también difiere: con el afásico, el busca reparar más la forma para lograr una adecuada cohesión, mientras que con el paciente con demencia utiliza reparaciones indirectas que buscan reparar la coherencia global. En una investigación sobre actividades de reformulación en pacientes afásicos brasileños, el análisis concluyó que estos movimientos discursivos constituyen un proceso de reconstrucción del sentido en la presencia de déficit lingüísticos (como parafasias, dificultades de acceso al léxico, etc.)^(8)^.

Martínez y Noemi^(15)^ analizaron las actividades de reformulación conversacional en tres grupos de participantes, los que se identificaron como controles, con deterioro cognitivo leve (DCL) y demencia tipo Alzheimer (DTA), y en una actividad de elicitación conversacional a partir de una historieta en imágenes. Se observó que los sujetos controles realizaban actividades de reformulación conversacional, adecuadas al curso de la conversación, a través de las correcciones sintácticas o léxicas y que las dificultades de acceso al léxico fue una de las causas de la necesidad de reformular. Por otra parte, los hablantes con DCL presentaron errores en el reconocimiento de personajes, acciones o motivaciones, inatingencias, incoherencias y dificultad para mantenerse en el tópico; lo que se destacó en este grupo es que no todas las actividades de reformulación cumplían con el objetivo de reparar lo dicho previamente para adecuar el discurso o de favorecer la comprensión mutua, aspectos esenciales en el desarrollo de una conversación, por lo que se puede evidenciar el efecto de la patología de base en la comunicación. En el grupo con DTA, las reformulaciones fueron menos frecuentes y ninguno de los enunciados reformuladores completó el objetivo de adecuar el discurso para la comprensión mutua. Los autores sugieren que la falta de actividades de reformulación puede deberse al descenso o ausencia de procedimientos de evaluación y automonitoreo metadiscursivos y que la capacidad de realizar auto-reformulaciones desciende en la medida en que declina el desempeño cognitivo. Una actividad frecuente en la interlocución con hablantes con DCL y DTA fue la hetero-reformulación. Este dato evidencia el hecho de que la adecuación del discurso conversacional, en estos casos, es de responsabilidad del interlocutor que necesita ser capacitado para tal.

Las reformulaciones realizadas por sujetos con afasia y con demencia en conversaciones en pareja fueron comparadas con foco en los aspectos interactivos y comunicativos^(16)^ y los resultados indicaron que las secuencias de reformulación fueron más frecuentes en aquellos con demencia además de evidenciar el rol activo de los interlocutores sanos en la resolución de problemas. Una investigación^(17)^ analizó los patrones secuenciales de comportamiento relacionados con la manifestación de las deficiencias en la gestión de temas y las conductas de facilitación en las interacciones cotidianas entre individuos con demencia y sus familiares y se observó que las dificultades para contribuir al tema de conversación eran dominantes en las personas con DTA y que los familiares aplicaban dos tipos de estrategias de reformulación: la primera ofreciendo inicio explícito de una reformulación, y la segunda, haciendo cambios de tema que funcionaban como puente hacia la vuelta al tópico del turno anterior.

Experiencias con personas mayores con distintas condiciones cognitivas permiten ejemplificar la utilidad de la evaluación orientada a la conversación bien como examinar la relación entre el diagnóstico, el desempeño cognitivo y las capacidades lingüísticas y conversacionales^(18)^. A través del Análisis Conversacional (AC) es posible observar una multiplicidad de elementos como el funcionamiento de la comunicación en un contexto real, de manera no idealizada pero basada en los datos, analizando el discurso conversacional por lo que contiene y no exclusivamente por lo que no contiene^(6)^. En este trabajo se pretende analizar, a través del AC, actividades de reformulación conversacional en personas mayores con y sin DTA, de manera tal, de dar cuenta si identificar este fenómeno en la evaluación de la persona con DTA es útil para su diagnóstico y/o su intervención.

## MÉTODO

Este estudio, se lleva a cabo bajo el enfoque de la metodología cu anti-cualitativa descriptiva. La orientación de descripción cualitativa centrase en el proceso más que en la obtención de resultados, por lo tanto, las conclusiones no son generalizables, sino más bien orientadas al fenómeno particular. La investigación fue aprobada por el comité de ética científico Centro Norte- UST nº142/2017, el 28 de febrero de 2018, asignando el código 153.17, y todos los participantes o sus representantes legales firmaron el consentimiento informado.

### Muestra

La muestra fue conformada por conveniencia, entre la población de adultos mayores particulares o residentes en establecimiento de larga estadía para adultos mayores (ELEAM) de la V Región de Chile, voluntariamente disponibles y accesibles y que cumpliesen con los criterios de inclusión y exclusión establecidos. Se seleccionaron 20 sujetos los cuales se dividieron en dos grupos: 10 en el grupo control (GC) y 10 en el grupo de usuarios con demencia de tipo Alzheimer (GDTA). Los criterios inclusión del GC fueron: ser mayor de 65 años, ambos géneros, presentar un nivel de instrucción de más de 4 años, usar un audífono en caso de presbiacusia leve a moderada, presentar un resultado en el MEEM e Pfeffer según la nota de corte descrita en las normas de los test y no presentar enfermedades neuropsiquiátricas. Los criterios de exclusión incluyeron: presentar enfermedades de tipo neurológicas o psiquiátricas, en distintos grados de severidad, presentar hipoacusia severa y consumir fármacos (psicotrópicos) que influyesen en el rendimiento de la evaluación cognitiva. Los criterios de inclusión para los sujetos del GDTA fueron: ser mayor de 65 años, ambos géneros, presentar un nivel de instrucción de más de 4 años, usar un audífono en caso de presbiacusia leve a moderada; tener diagnóstico médico neurológico o geriátrico actual de demencia tipo Alzheimer (DTA) en grado 5 según Escala de Deterioro Global (GDS) (demencia moderada), cumplir con los criterios del DSM-V para el trastorno neurocognitivo mayor y los criterios clínicos del National Institute of Aging (NIA) y/o los criterios clínicos centrales de DTA probable en los apartados A y C expuestos por la Alzheimer’s Association (AA). Fueron adoptados los siguientes criterios de exclusión: presentar enfermedades neurológicas de tipo accidente cerebro vascular o traumatismo craneoencefálico, psiquiátricas, evidencia de comorbilidad de otra enfermedad neurológica o no neurológica que afectase la cognición, consumir fármacos (psicotrópicos) que pudiesen influir en el rendimiento en la evaluación cognitiva y tener diagnóstico de hipoacusia severa.

Es necesario destacar que, en el análisis de la conversación cotidiana, a menudo es suficiente emplear una muestra con unos pocos textos, es decir, no más de diez entrevistas para no generar el fenómeno llamado ‘saturación teórica’ que da cuenta de la redundancia de la información en los datos, situación que no aporta significativamente a la reflexión teórica^(19)^.

### Procedimientos

A cada grupo se le aplicaron las siguientes pruebas neuropsicológicas para evaluar su estado cognitivo: Mini Mental State versión Chile (MMSE), Test de Boston Abreviado, Batería de evaluación Frontal (FAB), prueba de fluidez verbal fonológica FAS y semántica (animales y acciones), span de dígitos directos e inversos de Weschler, la prueba de memoria auditivo verbal del Rey y el Cuestionário Pfeffer. La técnica elegida para la toma de datos fue la elicitación clínica (clínical elicitation)^(20)^, que refiere que la situación de la conversación es provocada por el investigador para luego desarrollarla de manera lo más natural posible. Se utilizaron dos tareas conversacionales para generar un corpus, que se estimase como suficiente para cada sujeto, con dos tareas: tarea conversacional 1- entrevista semiestructurada de tipo autobiográfica, con preguntas abiertas que cada participante respondió desde su perspectiva, sin pistas informativas, según su ritmo y con adaptaciones naturales dentro del contexto discursivo; tarea conversacional 2 - compuesta por los siguientes ítems de la sección VI del Protocolo MetAphAs^(21)^: ítem 31, Descripción de un objeto o situación no presente; ítem 32, Desplazamiento temporal I - se le pide al participante que nos converse sobre lo que hizo el fin de semana; ítem 33, Desplazamiento temporal II: el participante debe contar sobre su primer trabajo, en qué consistía y qué es lo que hacía; ítem 34, Desplazamiento temporal III: se le pide al participante que cuente qué es lo que hará el próximo fin de semana o en las próximas vacaciones; ítem 35, Interpretar una escena; ítem 39, Capacidad para mentir: se le pide al participante que diga una mentira simple e ítem 40, Capacidad de ironizar: se le pide al participante que ironicen sobre algo a partir de un ejemplo. Se eligió el Protocolo MetAphAs porque es un instrumento de evaluación metalingüística que se relaciona con la naturaleza de la reformulación conversacional y su aplicación es de carácter ecológico, favoreciendo el desarrollo de un contexto conversacional natural.

Para el presente trabajo se utiliza el AC, una de las principales corrientes de la investigación etnometodológica^(22,23)^, cuyo enfoque es analizar datos empíricos de textos naturales con la finalidad de descubrir los mecanismos por medio de los cuales los actores otorgan significado a lo que les sucede, a lo que expresan y a lo que hacen ellos mismos. Los datos se transcribieron utilizando la lista de convenciones adaptadas por Tusón^(24)^ y las transcripciones se traspasaron al software AtlasTI versión 7, en donde se marcaron la presencia o ausencia de actividades de reformulación, los tipos de fuentes de problemas, tipos de reformulación y resultados de la actividad de reformulación conversacional. Se analizó además la cantidad, la frecuencia y el contexto lingüístico conversacional en que aparecen estas actividades.

Como no hay una forma única de realizar el procedimiento de análisis, se consideraron como paradigma de codificación, los siguientes aspectos: (a) codificación abierta: proceso analítico por medio del cual se identifican los conceptos y se descubren en los datos, sus propiedades y dimensiones y se codifica, es decir, se conceptualiza el código encontrado (por ejemplo, en el texto puede marcarse una pregunta del participante “¿qué?” la cual puede codificarse como “solicitud de aclaración” lo que significa que es una señal de petición de aclaración de lo que se está diciendo); (b) codificación axial: un proceso en el que se procede a clasificar los códigos encontrados en el proceso anterior, acomodándolos en categorías que favorecen el análisis de conexiones de sentido de los elementos verbales, sus consecuencias, derivaciones e interpretaciones con el objetivo de desmenuzar el fenómeno de la reformulación para encontrar características, reglas de uso, propiedades, etc.; (c) codificación selectiva: la última etapa en donde se integra y refina la teoría, sistematizando los datos que darán cuenta de los conocimientos elaborados a partir de la observación de actividades de reformulación y así responder a las preguntas de la investigación^(20)^. El corpus fue construido a partir de las conversaciones, sobre las cuales se realizó la codificación, la generación de conceptos y el desarrollo de explicaciones a partir de los mismos datos, que luego fueron contrastados con el marco teórico previo.

## RESULTADOS

A continuación, se presentan las características de los GC y GDTA en la Tabla 1.

**Tabla 1:** Características de los GC y GDTA según género, escolaridad y edad

	Código	Controles		DTA	
		Freq.	Percent.	Freq.	Percent.
**Género**	Masculino	3	30	2	20
	Femenino	7	70	8	80
**Escolaridad**	<8	2	20	2	20
	8- 12	7	70	6	60
	>12	1	10	2	10
**Edad**	66-69	1	10	1	10
	70-74	5	50	0	50
	75-79	2	20	1	20
	80 y +	2	20	8	80

Se puede verificar que los datos de los sujetos de los dos grupos son semejantes cuanto a género y escolaridad. En relación con la edad, 80% del GDTA tiene 80 y más años, y esta cifra es coherente con el hecho de que el DTA es una demencia que puede presentar inicio tardío y prolongarse por años^(25)^.

En la Tabla 2 se presentan los resultados de la evaluación neuropsicológica que se realizó en los sujetos de los dos grupos.

**Tabla 2:** Resultados de las pruebas neuropsicológicas de los participantes del grupo control y del grupo casos, media, desviación estándar y prueba T-Student

**Test**	**Grupo**	**N**	**M**	**DE**	**Prueba T-Student**
**MMSE**	**CONTROL**	10	27,30	2,983	0,000
	**DTA**	10	14,90	6,154	
**PFEFFER**	**CONTROL**	10	0	0	0,000
	**DTA**	10	22,70	7,072	
**FAB**	**CONTROL**	10	13,60	2,716	0,003
	**DTA**	10	8,10	4,149	
**FVSEM Animales**	**CONTROL**	10	11,40	3,658	0,001
	**DTA**	10	5,60	2,503	
**FVSEM Acciones**	**CONTROL**	10	10,50	5,968	0,032
	**DTA**	10	5,40	3,534	
**FVF-F**	**CONTROL**	10	8,90	3,315	0,000
	**DTA**	10	2,80	2,486	
**FVF-A**	**CONTROL**	10	8,60	4,351	0,021
	**DTA**	10	3,90	3,957	
**FVF-S**	**CONTROL**	10	8,20	2,741	0,013
	**DTA**	10	4,50	3,240	
**Ravalt- Trail 1**	**CONTROL**	10	4,20	0,789	0,010
	**DTA**	10	1,80	1,619	
**Ravalt- Trail 5**	**CONTROL**	10	7,10	3,178	0,030
	**DTA**	10	3,10	1,729	
**Ravalt- Post Intef.**	**CONTROL**	10	5,00	2,261	0,010
	**DTA**	10	1,80	1,317	
**Ravalt- Rec Diferido**	**CONTROL**	10	4,60	2,875	0,000
	**DTA**	10	0,50	0,972	
**Ravalt-Reconoc.**	**CONTROL**	10	7,80	1,476	0,000
	**DTA**	10	2,10	1,197	
**SPAN DD**	**CONTROL**	10	4,40	0,516	0,210
	**DTA**	10	3,90	1,101	
**SPAN DI**	**CONTROL**	10	3,30	0,675	0,027
	**DTA**	10	2,10	1,370	

Minimental (MMSE), cuestionario de Pfeffer, Batería de evaluación frontal (FAB), pruebas de fluencia verbal fonológica (FAS) y semántica (animales y acciones), Prueba de memoria auditivo verbal del Rey (RAVLT, ensayo 1, ensayo 5, post interferencia, recuerdo diferido y reconocimiento, Span de Dígitos Directos (Span DD) y Span de Dígitos Indirectos (SPAN ID).

Los datos demuestran que el GC logra puntajes más altos y con desempeño típico en relación con el GDTA, que presenta resultados que posicionan los sujetos como con deterioro cognitivo^(26-28)^. Cabe señalar que en relación con niveles de funcionalidad entregados pela escala de Pfeffer, el puntaje de cuatro de los participantes residentes en ELEAMs se presente más descendido probablemente porque por norma general no pueden cumplir con actividades diarias como cocinar y bañarse, por ejemplo, porque deben ser siempre asistidos independientemente de su nivel cognitivo o funcional. La prueba T-Student demuestra diferencia significativa en ambos grupos en todas las pruebas excepto en el span de dígitos en donde el grupo control alcanza un desempeño normal bajo.

Respecto al análisis del corpus, se procedió con el proceso de codificación abierta, y luego de codificación axial en donde se relacionaron categorías y subcategorías y se seleccionaron los códigos que se consideraron los más relevantes para conformar los focos temáticos de análisis. La Tabla 3 hace referencia a la cantidad de citas de reformulaciones y en ella se observa que los datos más díspares, entre los grupos, se relacionan con las reformulaciones inadecuadas. La Figura 1 expone la cantidad de citas de los tipos de reformulaciones inadecuadas.

**Tabla 3:** Cantidad de citas asociados a las auto-reformulaciones en grupo control y en grupo DTA.

**CÓDIGOS**	**CITAS CONTROL**	**CITAS DTA**	**TOTALES**
**Auto-reformulación auto-iniciada**	80	70	150
**Hetero-reformulación**	10	19	29
**Reformulación adecuada**	94	81	175
**Reformulación inadecuada**	1	28	29

**Figura 1:** Cantidad de numero de citas asociadas a reformulación inadecuada, en grupo control y en grupo DTA. Legenda: reformulación con errores de coherencia; reformulación non realizada frente a petición; reformulación incompleta; reformulación inadecuada que no responde a la pregunta; reformulación inadecuada con cambio de tópico: reformulación inadecuada; reformulación con errores menores; reformulación adecuada con información no verdadera

## DISCUSIÓN

A continuación, se presenta la descripción de los códigos más frecuentes y la discusión de su importancia en el contexto del estudio. El Cuadro 1 reúne los tipos de reformulaciones predominantes.

**Cuadro 1:** Tipos de reformulación

Auto-reformulación auto-iniciada
Hetero-reformulación
Reformulación adecuada
Reformulación inadecuada

En los ejemplos, cada uno de los hablantes aparecerá identificado con una ‘E’ que hace referencia al entrevistador y una ‘P’ que hace referencia al participante y una ‘A’ que hace referencia al acompañante; P asociado a un número sirve para identificar al participante y su entrevista, se agregará ‘S’ para señalar si es ‘sano’ y ‘D’ para señalar que se trata de un participante del GDTA y, se agregará al final un número que indica la posición de la cita en el texto.

### Auto-reformulación auto-iniciada

El análisis indicó que la auto-reformulación auto-iniciada fue el código más frecuente de todo el corpus, acumulando un total de 150 citas. De estas, 69 pertenecían a entrevistas del GDTA, mientras que 81 citas pertenecían a entrevistas del GC. El que este tipo de reformulación sea frecuente y dominante en todo el corpus coincide con lo encontrado en la literatura^(7,14,16)^.

Ejemplo 1:

P: Toda esa semana estuve donde mi nieto | entonces || tenía que venir a, **tenía que volver a la Ligua

(PS20: 078)

En este ejemplo, puede verse claramente la auto-reformulación auto-iniciada (el doble asterisco marca el inicio de la reformulación que reemplaza lo dicho previamente). En la siguiente cita, el sujeto claramente auto-reformula cambiando la expresión ‘para navidad’ por ‘víspera de navidad’

Ejemplo 2:

E: así lo hicieron? | ya y: ahora con quien vive usted?

P: no si yo vivo con nadie\

E: aquí con nadie?

P: claro

E: ya:

P: yo soy solita acá | un sobrino viene ahora como *para navidad*\ **víspera de navidad | vino a dejarme el regalo\

(PD2:95-100)

### Hetero-reformulación

La hetero-reformulación fue menos frecuente en comparación con la auto-reformulación. El total de citas fue de 29, y 20 de ellas correspondieron al GDTA, mientras que 9 al GC. La diferencia entre ambos grupos podría explicarse como un fenómeno asociado a la presencia de alteraciones lingüísticas, en donde el usuario con DTA requiere más apoyo por parte de su interlocutor y presenta más dificultad para realizar evaluaciones metalingüísticas, por lo que apoyarse en el interlocutor favorece la continuidad de la interacción conversacional^(2,12,13,29)^.

Podemos observar, en el ejemplo 3, las dificultades del usuario para contar cómo era su trabajo de niña, que consistía en entregar almuerzos a trabajadores. El interlocutor es quien resume lo dicho para facilitar el entendimiento mutuo y la progresión de la conversación.

Ejemplo 3:

P: a los trabajadores para ir a llevar a a una panadería ósea a una farmacia que había con: *era uno primero el doce después el uno que era otro grupo y así \ hasta la 1 y media*

E: **ya\ a varios grupos por turno\ (PD6:027-028)

Otro elemento que surge en esta investigación es aquel marcado con el código ‘reafirmación de la reformulación’, que es una acción que cierra la hetero-reformulación y que cumple una función no menos relevante. Si bien, no es tan frecuente, su aparición fue más o menos similar en ambos grupos. Como fenómeno puede dar cuenta de la conexión que tiene el hablante con lo que se está diciendo o al menos indicar a través de su aceptación que sigue presente en la interacción comunicativa^(7,15)^. El código tuvo 11 citas y 5 de ellas corresponden a usuarios con DTA.

En el ejemplo 4, la participante con DTA debe mencionar la ciudad en la que vivía y se equivoca mencionando una de las calles principales. Después de que el entrevistador hetero-reformula, la participante acepta y valida la corrección y a partir de ahí puede continuar con lo que estaba diciendo.

Ejemplo 4:

P: *en Condell* que estaba en ese tiempo

E: **en Valparaíso

P: *Si eso es | Después vivíamos ahí en Valparaíso*

(PD4:108-110)

A seguir, se ofrece el ejemplo del corpus de una participante con DTA con GDS 5, cuyo discurso, muchas veces ininteligible, revela diversas marcas de faltas a la coherencia, no logrando presentar conexión con nuevos tópicos. Ella trata de contar sobre su primer trabajo y cuando intenta decir que su abuela preparaba almuerzos, ella dice ‘arreglaba’ - la entrevistadora reformula diciendo ‘los preparaba’ y ella responde diciendo ‘claro’. Su afirmación no necesariamente indica que comprendió en contexto de la intervención de E, sino más bien señala que ella marca su presencia en la conversación. Es interesante notar que, al responder, elle revela que su capacidad de interactuar conversacionalmente está preservada.

Ejemplo 5:

E: ya: y después la primera vez que trabajo?

P: iba a llevar la vianda al colegio\ | pero después me subía las | las patas que tenía que devolver

E: ya: | usted le daba comida\

P: no: yo no lo daba mi abuelita le daba todo |

E: ya:\

P: | ósea ella le: *| arreglaba*

E: **=los preparaba=

P: claro

(PD14:043-049)

### Tipos de enunciados reformuladores

En el corpus se marcaron un total de 204 citas divididas en distintos tipos de enunciados reformuladores, resultado ya verificado en la literatura^(14,15,17)^. A continuación, se describen los tres tipos más frecuentes.

-*Enunciado reformulador de tipo ampliación sintáctica y semántica* es el que agrega información por una extensión sintáctica y semántica en comparación con el enunciado fuente. En este código se concentran 42 citas en total: 19 de ellas corresponden a entrevistas del GDTA y 23 del GC.

Ejemplo 6:

P: yo *estuve* bueno **cuando estuve en el regimiento estuve en punta arenas

(PD1:025)

En este ejemplo, el enunciado reformulador repite ‘estuve’ y agrega más información expresada en un enunciado con más elementos sintácticos y semánticos, de manera tal que completa información en relación con lo que quiere contar el participante.

En el próximo recorte, la participante agrega en el enunciado reformulador más información en comparación con el enunciado fuente.

Ejemplo 7:

P: (…) | y *yo me vengo para acá* | **el sábado me vendría para acá y me vengo a mi casita | (…)

(PS20:052)

La leve diferencia de cantidad de citas para este código podría sugerir que los participantes sanos tienen más condición de ampliar lingüísticamente la información que los participantes con DTA ya que la actividad metalingüística, parte de la metacognición, regula la posibilidad lingüística para hacer uso de los subcódigos oral y escrito^(17)^.

-*Enunciado reformulador de corrección informativa*. Este código incluye 51 citas de las cuales 36 corresponden a las entrevistas del GDTA y 15 corresponden a entrevistas de personas sanas.

Ejemplo 8:

E: y usted con quien vive ahora?

P: | yo vivo sola\

E: *acá*?

P: **no po acá vivo con toda la gente

(PD5:105-108)

En este ejemplo la participante con DTA se equivoca en decir con quien vive. Si bien corrige a través de un procedimiento de reformulación, el nuevo enunciado no es tan preciso pues no informa sobre el hecho de que vive en un ELEAM. En el siguiente ejemplo, podemos ver como el participante sano corrige lo dicho anteriormente para dar sentido a la historia que está contando.

Ejemplo 9:

P (…) | y actualmente, *no actualmente*, **como hace 25 años, tenía un tremendo negocio en Viña (…)

(PS23:396)

Con este código puede apreciarse una diferencia importante en la cantidad de citas de cada grupo. Los participantes con DTA aparentemente tienen más necesidades de corregir enunciados que los participantes sanos por razones de problemas cognitivos^(2,14,15,18)^.

### *Enunciado reformulador de precisión informativa*. Este código acumuló 50 citas de las cuales 12 citas corresponden a personas con DTA, mientas que 38 citas corresponden al GC

Ejemplo 10:

P: | *yo tenía mi casa* | vivían en la casa de 2 pisos\

E: m:

P: ósea **que esta arrendado la parte de abajo y en arriba vivía yo (…)

(PD2:024-026)

Ejemplo 11:

P: o sea puras circunstancias peligrosas y | y el papá estaba lavando la loza | está secando un vaso | pero él tiene un | lleno de espuma *el lava* | **el lava plato

(PS22:029)

En el caso del ejemplo 10, la hablante reformula para hacer más precisa la información sobre dónde vivía antes de entrar al ELEAM. En el ejemplo 11 la hablante reformula para hacer más precisa la información en el contexto de descripción de una lámina.

La notable diferencia en la cantidad de citas de ambos grupos sugiere que para el GD la reformulación tiene la finalidad de agregar mayor precisión en lo que quiere decir, para darse a entender mejor. Si además tomamos como referencia el código reformulación de corrección informativa, veremos que los participantes con DTA se ocupan más en corregir que en ser más precisos. La preocupación por la precisión es una preocupación más retórica, indicando que el hablante está constantemente monitoreando y colaborando a la comprensión mutua, hecho que se reduce en los hablantes con DTA^(12-15)^.

### Reformulación adecuada

La reformulación adecuada es aquella en la que se evidencia la función hacer el enunciado más entendible y aceptable y facilitar la comprensión mutua. Con este código, se pretendió valorar la calidad de la reformulación en cuanto a si repara o no, favorece o no, la comprensión mutua. Se marcaron en total 175 citas, de las cuales 83 pertenecen a participantes con DTA y 92 corresponden a participantes control. Cabe mencionar que las 92 citas corresponden al total de reformulaciones observadas en los sujetos sanos, es decir, todas las reformulaciones (auto-reformulación y hetero-reformulación) que los sujetos sanos realizaron, fueron adecuadas.

Ejemplo 12:

P: pero *conocí* la: | la **el lugar donde nació mi padre punta arenas era puntarenense

(PD1:025)

Ejemplo 13:

P: no, no lo que pasa es que mi señora *se me enf*:: **se me cayó aquí en el dormitorio y se quebró la cadera

(PS23:035)

En el ejemplo la reformulación adecua lo que dice al interlocutor de manera efectiva. Lo mismo puede verse en el ejemplo 13 sacado de una entrevista con un participante sano.

En el corpus de la muestra se observan varias actividades de reformulación y la diferencia principal entre los grupos está en que la cantidad de actividades de reformulación en el GDTA es mayor y que no todas sus reformulaciones son adecuadas. Las reformulaciones que fueron marcadas como adecuadas eran similares a las del GC, sin embargo, los motivos para reformular variaron porque, en la interacción conversacional, aparecen dificultades de acceso al léxico, problemas de memoria y otras alteraciones propias del DTA y que motivan a reformular. También es necesario considerar que un 23% de las reformulaciones adecuadas en el GDTA se debe a hetero reformulaciones en donde la responsabilidad de adecuar lo que se dice recae en el interlocutor sano.

### Reformulación inadecuada

La reformulación inadecuada es aquella que los hablantes hacen, pero se observa que no se logra reparar, aclarar o mejorar lo que se está diciendo. El total de citas fueron 27 y todas se verificaron en las entrevistas con participantes del GDTA.

Ejemplo 14:

P: (…) mi abuela me tenía listo *yo salía de la playa* (gesto con cabeza de error) ósea **de la playa de la escuela (…)

(PD6:62)

En este ejemplo la participante intenta contar sobre el momento en que iniciaba su labor, ‘cuando salía de la escuela’, entonces aparece una parafasia ‘playa’, ella logra darse cuenta de su error, inicia una auto-reformulación, pero no logra omitir de su enunciado la palabra ‘playa’.

Un enunciado reformulador suele aparecer en el discurso señalado con un marcador o indicador (por ejemplo: o sea, es decir, más bien, digo, etc.), lo que permite establecer una relación semántica con el enunciado fuente^15^. De este modo, los procedimientos reformuladores aparecen luego de que el hablante ha evaluado la expresión previa como insuficiente, inapropiada o insatisfactoria. Un aspecto relevante observado en los hablantes GC es que el resultado de la actividad de reformulación conversacional se identificó como adecuada, que es el objetivo principal de cualquier tipo de reparación conversacional. Tal procedimiento, fue menos frecuente o simplemente no ocurrió en los hablantes del grupo GDTA que, si bien realizó actividades de reformulación de característica similar a los sujetos del GC, en su mayoría lo hizo también por otro tipo de dificultades, dentro de las cuales se mencionan las dificultades de acceso al léxico, elaboración de enunciados, fuentes incoherentes, parafasias, errores en sus narraciones y por la marcada dificultad para mantenerse en el tópico. Este desempeño está en acuerdo con lo descrito en la literatura sobre bajo desempeño cognitivo como factor de dificultades lingüísticas (que a su vez generan actividades de reformulación conversacional)^(14,15,18,29,30)^.

La presencia de enunciados reformuladores que resultaron inadecuados o que no cumplían con el objetivo de ajustar el discurso o de favorecer la comprensión mutua caracteriza el grado de adecuación del GDTA. Estos datos confirman las conclusiones de autores^(12-16,29)^ quienes encontraron que en personas con trastorno cognitivo leve y demencia se reduce la capacidad de realizar reformulaciones adecuadas al contexto. La adecuación pudo conseguirse mayormente, en el GDTA, con las hetero-reformulaciones, las que, al estar a cargo de un interlocutor sano, posibilitan la reparación del sentido de lo que se está diciendo. La reducción de auto-reformulación observada en este grupo puede deberse al descenso o ausencia de procedimientos de evaluación metadiscursiva^(16,18)^, que se relaciona con el monitoreo cognitivo de los hablantes durante la conversación^(18)^. Por lo tanto, al tener un desempeño cognitivo reducido, la capacidad de reformulación se reduce o se hace nula.

En resumen, la auto-reformulación auto-iniciada fue observada en menor escala y la hetero-reformulación en mayor proporción en los sujetos con DTA cuando comparados con los sujetos sanos. En las reformulaciones analizadas en el GDTA, se observaron frecuentes dificultades de acceso al léxico, de dificultades de memoria y dificultades de comprensión. En termino de actividades de reformulación, se pudieron observar, a consecuencia de esto, enunciados fuentes con errores, imprecisos o incompletos.

El 70% del GC se encuentra entre los 70 y 79 años evidenciando una diferencia significativa entre ambos grupos. Con este resultado, se reconoce que la diferencia de edad podría tener algunas implicancias en relación al desempeño cognitivo y conversacional, bajo la premisa de que el envejecimiento genera cambios cognitivos y comunicativos en los sujetos mayores. Sin embargo, es importante tener presente que la edad no es un reflejo proporcional al grado de deterioro que pueda tener una persona, ya que el envejecimiento se caracteriza por su importante variabilidad inter-sujeto. Por esta razón, a través de las pruebas neuropsicológicas obtenemos puntajes que, de acuerdo con sus propias normas, es posible interpretar el real estado cognitivo de una persona mayor, datos que permiten la comparación entre sujetos y entre grupos de sujetos. Esta información no sería interpretable a partir de la edad misma. En relación con las implicancias a nivel conversacional, a la fecha existe muy poca evidencia sobre la relación entre la edad y la capacidad de reformular (Martínez y Noemi, 2016; Mac-Kay, et al, 2017). Por esta razón, en este estudio, los principales hallazgos se relacionan principalmente al nivel cognitivo de los participantes, teniendo presente que la edad puede ser un factor para analizar en futuras investigaciones sobre el desempeño conversacional en adultos mayores.

## CONCLUSION

En la presente investigación se ha podido evidenciar que esta capacidad de reparación conversacional es sensible al desempeño cognitivo de personas con DTA. El grado de adecuación es el principal factor de diferencia entre ambos grupos. Los datos señalaron un descenso de la capacidad de reformulación, evidenciado en los hablantes con DTA que se manifiesta con reformulaciones que no cumplían con el objetivo de corregir o adecuar el discurso, y que resultaron inadecuadas. Otro resultado importante es el relacionado con la funcionalidad de la interacción conversacional, porque ésta no necesariamente se sostuvo en el tiempo debido en el discurso del GDTA. Es necesario resaltar que el interlocutor juega un rol importante en la díada, procurando compensar las limitaciones de la persona con demencia.

Se destaca la importancia de futuros estudios sobre habilidades conversacionales y actividades de reformulación en el contexto de la persona mayor sana y en aquellas con patologías que afectan las funciones cognitivo-comunicativas.
